# Supplementary material for: Technology-based Health Solutions for Cancer Caregivers to Better Shoulder the Impact of COVID-19: A Systematic Review Protocol
Source: Res Sq. 2021 Jan 19:rs.3.rs-66218. Originally published 2020 Sep 1. Preprint. [Version 2] doi: 10.21203/rs.3.rs-66218/v2 (PMC7480034; doi:10.21203/rs.3.rs-66218/v2)
Supplement: Supplement 1 [file NIHPPRS66218v2-supplement-1.pdf]

Supplementary Files

This is a list of supplementary files associated with this preprint. Click to download.

- PRISMAPchecklist.pdf
